# Concordance of *KRAS/BRAF* Mutation Status in Metastatic Colorectal Cancer before and after Anti-EGFR Therapy

**DOI:** 10.1155/2009/831626

**Published:** 2010-03-10

**Authors:** S. Gattenlöhner, B. Etschmann, V. Kunzmann, A. Thalheimer, M. Hack, G. Kleber, H. Einsele, C. Germer, H. -K. Müller-Hermelink

**Affiliations:** ^1^Institute of Pathology, University of Würzburg, 97080 Würzburg, Germany; ^2^Department of Internal Medicine II, University of Würzburg, 97080 Würzburg, Germany; ^3^Department of Surgery, University of Würzburg, 97080 Würzburg, Germany; ^4^Institute of Pathology, Ostalb-Klinikum Aalen, 73430 Aalen, Germany; ^5^Department of Internal Medicine, Ostalb-Klinikum Aalen, 73430 Aalen, Germany

## Abstract

Anti-EGFR targeted therapy is a potent strategy in the treatment of metastatic colorectal cancer (mCRC) but activating mutations in the *KRAS* gene are associated with poor response to this treatment. Therefore, *KRAS* mutation analysis is employed in the selection of patients for EGFR-targeted therapy and various studies have shown a high concordance between the mutation status in primary CRC and corresponding metastases. However, although development of therapy related resistance occurs also in the context of novel drugs such as tyrosine kinase-inhibitors the effect of the anti-EGFR treatment on the *KRAS/BRAF* mutation status itself in recurrent mCRC has not yet been clarified. Therefore, we analyzed 21 mCRCs before/after anti-EGFR therapy and found a pre-/posttherapeutic concordance of the *KRAS/BRAF* mutation status in 20 of the 21 cases examined. In the one discordant case, further analyses revealed that a tumor mosaicism or multiple primary tumors were present, indicating that anti-EGFR therapy has no influence on *KRAS/BRAF* mutation status in mCRC. Moreover, as the preselection of patients with a *K* *R* *A* *S* ^*wt*^ genotype for anti-EGFR therapy has become a standard procedure, sample sets such ours might be the basis for future studies addressing the identification of potential anti-EGFR therapy induced genetic alterations apart from *KRAS/BRAF* mutations.

## 1. Introduction

**Figure 1:**
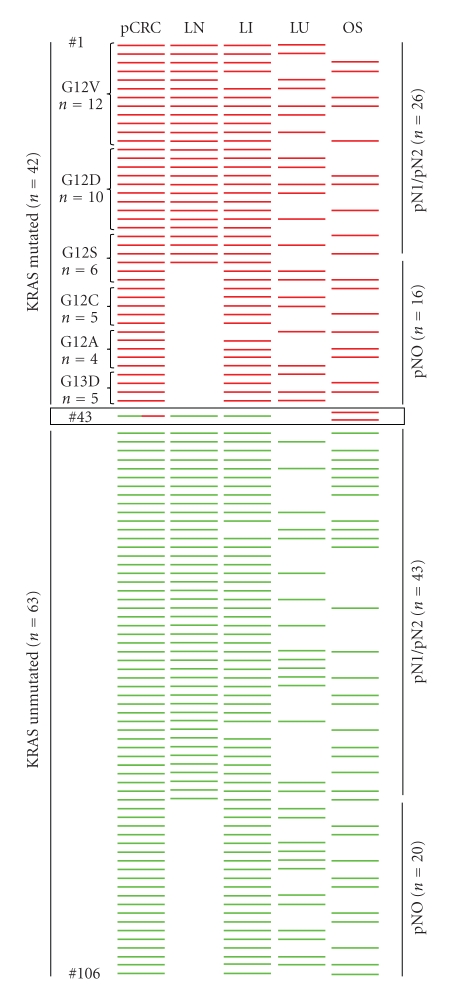
Overview of patient data from an investigation of the concordance of 106 primary CRCs and 270 corresponding metastases syn-/metachronic metastases. Each line represents an individual patient with primary CRC and metastatic manifestations. Red bars demonstrate *KRAS* mutation positive CRCs (*n* = 42) with corresponding lymph node metastases (*n* = 26), liver metastases (*n* = 40), lung metastases (*n* = 22), and other sites (*n* = 18) including bone marrow (*n* = 6), soft tissue (*n* = 5), and peritoneum (*n* = 7). Green bars show *KRAS* mutation negative CRCs (*n* = 63) with corresponding lymph node metastases (*n* = 43), liver metastases (*n* = 61), lung metastases (*n* = 28), and other sites (OS) (*n* = 32) including bone marrow (*n* = 10), soft tissue (*n* = 13) and peritoneum (*n* = 9). In case #43 a heterogeneously differentiated primary CRC (see also Figure 2) showed a *KRAS* mosaicism (red and green bars in pCRC) with detection of *KRAS* mutation G12V in other sites (OS, undifferentiated soft tissue und peritoneal metastases (red bars)) but not in moderately differentiated lymph node and liver metastases (LN and LI, green bars). LN: lymph node; LI: liver; LU: lung; OS: other sites.

**Figure 2:**
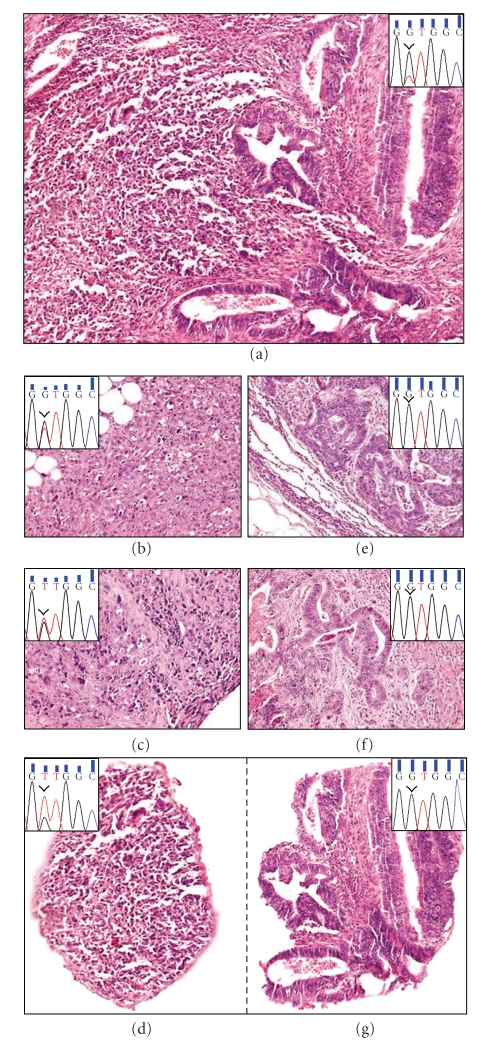
Morphological changes and results of *KRAS* mutation analyses in case #43 of a heterogeneously differentiated CRC with mosaicism for *KRAS* mutation G12D. In Figure 2(a) moderately (right half) and undifferentiated (left half) tumor areas reveal positive detection of *KRAS *mutation G12V (inset). In contrast, only in soft tissue (b) and peritoneal (c) metastases harbouring exclusively undifferentiated tumor infiltrates an identical *KRAS* mutation was detectable ((b) and (c) insets), whereas in lymph node (e) and liver (f) metastases with moderately differentiated tumor infiltrates only, no *KRAS* mutation was found ((e) and (f) insets). After microdissection of undifferentiated (d) and moderately differentiated (g) areas from primary CRC the *KRAS* mutation G12V was only detectable in the undifferentiated fraction ((d), inset). Images were produced with a B × 50 microscope (Olympus, Hamburg, Germany) and a DP50 digital camera with DP-Soft 5.0 software (Olympus, Hamburg, Germany).

Colorectal carcinoma (CRC) is one of the most common forms of malignant neoplasia and frequently takes a fatal course following metastasis [1]. CRC is a multipathway disease involving dysregulatory phenomena in a number of signal transduction pathways [2]. The epidermal growth factor receptor (EGFR), a tyrosine kinase receptor belonging to the ErbB family, is overexpressed in 25%–80% of CRCs and has been found to play a major role in the pathogenesis of CRC by inducing downstream signaling pathways such as the phosphatidylinositol-3-kinase/Akt and Ras/Raf/mitogen-activated protein kinase (MAPK) pathways, which are crucial in the regulation of cell growth, proliferation, apoptosis, invasion, migration, and angiogenesis [3]. Consequently, antibodies targeting EGFR, such as cetuximab and panitumumab, have been examined for therapeutic efficacy in CRC patients [4]. Although it was determined that combination therapy of irinotecan and cetuximab is significantly more successful in the treatment of metastatic CRC (mCRC) than irinotecan alone, the overall therapeutic response rate to combined cetuximab therapy is less than 30%, suggesting that there are escape mechanisms present in many cases of CRC [5, 6]. Among others, mutation of the genes encoding the Kirsten rat sarcoma viral oncogene homologue (*KRAS*) and the V-raf murine sarcoma viral oncogene homolog B1 (*BRAF*) wes established as two of these mechanisms and preselection of CRC patients with a *K* *R* *A* *S* ^*wt*^ genotype have been shown to increase the therapeutic efficacy of anti-EGFR therapy [7, 8]. Therefore, clinical trials involving anti-EGFR therapy are now commonly conducted with patients preselected for *K* *R* *A* *S* ^*wt*^ mutation status [9, 10]. To ensure that therapy targeting EGFR is effective in primary CRC as well as in corresponding metastases, various studies have examined the concordance or discordance of *KRAS* and *BRAF* mutation status in primary CRC and corresponding metastases. Although the results of these studies appear contradictory in part, the majority of authors report high rates of concordance between the mutation status of *KRAS* in primary tumors and corresponding metastases [4, 9, 11–16]. In a study published recently by our group, *KRAS* mutation status was monitored in the primary tumors and corresponding metastases of 106 cases of mCRC [17]. Here, we found concordance in the mutation status of *KRAS* in 105 of 106 cases (Figure 1) and were able to show that the only case of discordance was due to a tumor mosaic or the coexistence of multiple primary tumors (Figure 2), a fact that could help in explaining the partially contradicting results reported in the past [13, 17]. Moreover, in analogy to other types of cancer [18–20], therapy-related resistance based on a treatment-induced shift in *KRAS* and/or *BRAF* mutation status could also play a role in explaining the low therapeutic efficacy of anti-EGFR therapy in mCRC by rendering tumor cells initially responsive to anti-EGFR mAbs resistant to this therapeutic regimen [9]. 

## 2. Materials and Methods

49 individual specimens of 21 metastatic CRCs and corresponding metastases collected before and after combined therapy with cetuximab were examined using CGH, certified PCR/DNA sequencing protocols (*KRAS* exon 2, Gl12/13; *BRAF* exon 15, V600E) as well as allele-specific PCR [8]. 

The majority of samples analyzed in this approach derived from the sample pool of 106 mCRC with 270 syn-/metachronic metastases used in our earlier study on the concordance of *KRAS* mutation status in primary CRC and corresponding metastases [17]. Biopsy sets were collected before and after combined cetuximab therapy, whereby the samples collected before therapy were taken from primary CRCs and/or liver metastases, while those gathered after therapy were from metastases in different locations, predominantly the liver (Table 2). Each CRC studied was clinically documented as a single primary malignant tumor in the colon/rectum and all metastases were identified as such by a characteristic “CRC-like” immunohistochemical profile (cytokeratin 20 positive, cytokeratin 7 negative). Following pathohistological characterization, tumor cells were enriched to >90% from 4–6 10 *μ*m slices using microdissection. Two independent samples from each specimen were incubated overnight in lysis buffer containing proteinase K at 56°C and DNA was subsequently extracted by column affinity chromatography (Qiagen DNA Minikit, Cat No 51306). 

CGH was performed as previously described [17]. Briefly, DNA was labeled by nick translation with biotin-16-dUTP (Roche Diagnostics, Mannheim, Germany). After inactivation of DNase I (Roche Diagnostics, Mannheim, Germany) equal amounts (1 *μ*g) of tumor and reference DNA (DIG-labeled DNA from placental tissue of a healthy newborn) were cohybridized on metaphase slides (Vysis, Downers Grove, IL). Signals were visualized with a Zeiss Axiophot fluorescence microscope and analyzed with the ISIS digital image analysis system (MetaSystems, Altlussheim, Germany). 

*KRAS* and *BRAF* mutation analyses were performed on two independent samples from all primary tumors/metastases using protocols described previously [8]. Briefly, DNA was amplified using Taq DNA Polymerase (Invitrogen Cat. No. 10342-020) and allele specific primers (Eurofins, see Table 1). Amplificates were visualized in 2% agarose gels and purified from the gels using column affinity chromatography (QIAquick Gel Extraction Kit, Qiagen Cat. No. 28704). Sequencing PCR was performed using PCR primers (see Table 1) and ABI BigDye Terminator v3.1 Cycle Sequencing RR-100 (ABI Heidelberg, Germany) as described [17].

## 3. Results

In the first part of our study we analyzed 106 metastatic CRCs with at least 2 multifocal and syn-/metachronic metastases (*n* = 270) using PCR/DNA sequencing protocols certified by the German Society for Pathology (exon 2, Glycin12 and Glycin13) as well as allele-specific PCR approaches. As shown in Figure 1, we observed concordance of the *KRAS* mutation status between primary CRCs and all corresponding metastases in 105 of 106 patients. However in one case (Figure 1 #43) of a *KRAS* mutation G12V positive moderately differentiated and undifferentiated primary CRC (Figure 2(a)), the mutation was detectable in soft tissue and peritoneal metastases with infiltrates from the undifferentiated tumour fraction (Figures 2(b) and 2(c)), but not in moderately differentiated lymph node and liver metastases (Figures 2(e) and 2(f)). Microdissected subfractions of the primary heterogeneous CRC showed a corresponding mutational mosaicism with detection of *KRAS* mutation G12V only in the undifferentiated tumour areas (Figures 2(d) and 2(g)). 

In the second part of the study we addressed the question, whether combined cetuximab therapy might influence the *KRAS*/BRAF mutation status in mCRC. Therefore 16 patients from the described sample pool (Figure 1) as well as 5 novel patients (including case #4, see Table 2) suffering from mCRC were analyzed for the *KRAS*/BRAF mutation status using the above mentioned molecular and immunohistochemical techniques. 

We hereby observed a concordance in the *KRAS* and *BRAF* mutation status between samples from primary CRCs and/or corresponding metastases before and after combined cetuximab therapy in 20 of 21 patients (Table 2). The timespan between first and last sampling ranged between 4 and 81 months (average 25,8 months) and the patients involved in the study received 1 to 5 rounds of therapy (average 1,8 rounds) during this period. 

**Figure 3:**
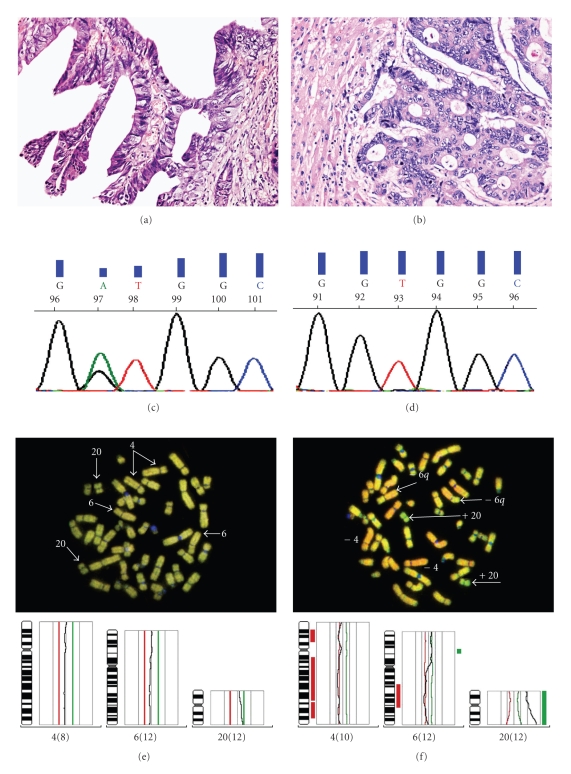
Further analysis of biopsies from the patient with a discordant *KRAS* mutation status before and after anti-EGFR therapy (#4, see Table 2) showing varying morphology between primary CRC with papillary tissue organization (a) and liver metastasis with tubular tissue organization (b). *KRAS* mutations analysis of corresponding specimens demonstrate detection of *KRAS* mutation exon 2 Gly12D (c) in primary CRC whereas an unmutated *KRAS* status in the liver metastasis was detectable (d). In fluorescence scan and data analysis of CGH only in the liver metastasis biopsied after treatment (f) but not in the primary CRC (e) a decrease in fluorescence of chromosomes 4 and 6 and an increase of chromosome 20 (arrows) could be seen.

In the one case of discordance between *KRAS* mutation status before and after combined cetuximab therapy (case #4), a mutated *KRAS* gene (Exon 2 G12D) was found in the primary CRC, while no *KRAS* mutation was observed in a liver metastasis sample obtained after combined cetuximab therapy (Table 2). Further analysis of the biopsy samples in this patient revealed that the primary CRC sampled before therapy and the liver metastasis biopsied after therapy showed differences not only in their *KRAS* mutation status but also in morphology and overall genetic composition (Figure 3). While the primary tumor was predominantly organized in a papillary fashion (Figure 3(a)), the liver metastasis displayed a distinctly tubular organization (Figure 3(b)). As mentioned above, microdissected samples gathered from each of these specimens showed distinct genetic sequences in exon 2 of the *KRAS* gene. While a mutated *KRAS* gene (Exon 2 G12D) was observed in the primary CRC (Figure 3(c)), a *K* *R* *A* *S* ^*wt*^ genotype was found in the liver metastasis (Figure 3(d)). Moreover, comparative genomic hybridization performed on the samples revealed that each of these displayed different genetic alterations. In comparison to the primary CRC (Figure 3(e)), samples from the liver metastasis showed sequence losses in chromosomes 4 and 6, while gains were observed in chromosome 20 (Figure 3(f)).

## 4. Discussion

Development of therapy-related resistance is a frequent phenomenon in cancer and also occurs in the context of novel drugs such as monoclonal antibodies and tyrosine kinase inhibitors. In gastrointestinal stromal tumors (GISTs), the most common mesenchymal neoplasm of the gastrointestinal tract, mechanisms of resistance to imatinib mesylate (Gleevec^R^) include both de novo and, more frequently, acquired resistance, which may occur after several months of drug administration and most often is based upon an acquired second mutation in the c-kit and PDGFR*α* genes [18, 19]. In B-cell lymphoma, resistance to the chimeric anti-CD20 monoclonal antibody rituximab, the first monoclonal antibody to have been registered for the treatment of B-cell lymphomas, is suggested to be due to reduced expression of CD20, the failure of rituximab to trigger the cells leading to inhibition of antibody-dependent and complement-dependent cell toxicity (ADCC and CDC), as well as hyperactivation of antiapoptotic signaling pathways such as p38 MAP kinase, NF-kappaB, ERK1, and AKT [20]. 

In the first part of our study we analyzed a well-documented cohort of 106 metastatic CRCs with 270 syn-/metachronic metastases concerning the *KRAS* mutation status showing an overall concordance of the *KRAS* mutation status in primary CRCs and metastases in 99% of the cases examined that stands in good accordance to previous reports [21]. However, apart from potentially coexisting secondary malignancies, the *KRAS* mutation mosaicism found in one heterogeneously differentiated CRC (#43, Figures 1 and 2) may explain the discordant results concerning this trait in primary CRCs and metastases reported previously [12, 13, 22] and underlines the necessity for diligent clinical and histological characterization of any atypical tumour manifestation in mCRC to prevent misleading results with negative impact on anti-EGFR targeted therapies. 

The second and major part of our study was to investigate whether therapy-related resistance due to acquired second *KRAS/BRAF* mutations also occurs in metastatic colorectal cancer after anti-EGFR therapy. Therefore we analyzed 49 individual specimens from 21 mCRC collected before and after combined cetuximab therapy. Of the 21 patients examined in this study, 20 showed concordance between the *KRAS* mutation status before and after combined cetuximab therapy, while the rate of concordance for *BRAF* was 100%. In one patient (case #4, Table 2) that did not derive from the above mentioned sample pool of 106 mCRC, the *KRAS* mutation status was discordant between the samples collected before and after cetuximab therapy, but due to further analysis of these samples with clearly different morphological and genomic features (Figure 3) it might be suggested that populations of carcinoma cells heterogeneous with respect to wild-type and mutant *KRAS* were probably present in the primary carcinoma, but the metastatic clone derived from a *KRAS* negative population, as reported previously [22, 23]. 

The results of this study provide first evidence that secondary *KRAS*/BRAF mutations do not play a major role in therapy-related resistance to anti-EGFR antibody treatment in mCRC, although it cannot be excluded that *KRAS*/BRAF mutations beyond Glycin12/13 *KRAS* exon2 and V600E BRAF exon15 as well as secondary resistance due to combinational chemotherapies (as in most patients investigated) are responsible for therapy-related resistance in mCRC. Moreover, as the preselection of patients with a *K* *R* *A* *S* ^*wt*^ genotype for cetuximab therapy has become a standard procedure, sample sets such as the one used in this study will have to be conserved carefully for use in future studies particularly with respect to analyses addressing the identification of anti-EGFR therapy-induced genetic alterations apart from *KRAS*/*BRAF* mutations.

## Figures and Tables

**Table 1:** Primer data for sequencing and allele specific PCR for *KRAS* exon 2 and *BRAF* exon 15 mutation analysis.

*KRAS*	

*d*1	5′-GAG TTT GTA TTA AAA GGT ACT GG-3′
*d*2	5′-TAC TGG TGG AGT ATT TGA TAG TG-3′
*r*1 + *r*2	5′-CTG TAT CAA AGA ATG GTC CTG-3′

BRAF	

*d*	5′-TGC TTG CTC TGA TAG GAA AAT-3′
*r*	5′-CTG ATG GGA CCC ACT CCA T-3′

**Table 2:** Overview of the clinical patient data. 20 of 21 patients show concordance of the *KRAS* (exon 2, Gly 12/13) and *BRAF* (exon 15, V600E) mutation status between samples of primary CRCs and/or corresponding metastases before and after combined cetuximab therapy. In one case (#4), the primary CRC had a mutated *KRAS* gene (Gly12Asp), while the liver metastasis biopsied after combined cetuximab therapy showed a *K* *R* *A* *S* ^*wt*^ genotype. *BRAF* mutation status in this case was concordant between the samples gathered before and after anti-EGFR therapy.

Case no.	Sex/Age	Date and localisation of tumor manifestaion	*KRAS* Gly12/13 (exon2)	BRAF V600E (exon15)	Anti-EGFR therapy
1	M/51 y	09/05 primary CRC	Gly12Val	WT	01/07–04/07 Folfiri/Cetuximab (PD)
		11/07 small bowel metastasis	Gly12Val	WT	dead 05/08

2	M/71 y	11/02 soft tissue	Gly12Cys	WT	09/04–08/05 Folfiri/Cetuximab (PR)
		03/06 mesocolon transversum metastasis	Gly12Cys	WT	

3	M/46 y	03/07 primary CRC	WT	WT	08/07–06/08 Folfiri/Cetuximab (PR)
		02/09 peritoneal carcinosis	WT	WT	

4	M/68 y	11/06 primary CRC	Gly12Asp	WT	04/07–02/08 Fufox/Cetuximab(PR)
		03/08 liver metastasis	WT	WT	

5	F/56 y	07/08 primary CRC	Gly12Asp	WT	07/08–09/08 Fufox/Cetuximab (PD)
		12/08 peritoneal carcinosis	Gly12Asp	WT	dead 12/08

6	M/71 y	12/05 primary CRC	Gly12Val	WT	01/06–07/06 Folfiri/Cetuximab (PD)
		12/06 liver metastasis	Gly12Val	WT	dead 04/08

7	F/58 y	01/08 primary CRC	WT	WT	04/08–07/08 Folfiri/Cetuximab (PD)
		07/08 peritoneal carcinosis	WT	WT	dead 07/08

8	F/44 y	11/07 primary CRC	WT	WT	05/08–03/09 Folfox/Cetuximab (PR)
		12/08 peritoneal carcinosis	WT	WT	

9	F/66 y	02/04 primary CRC	Gly12Ser	WT	07/06–09/06 Folfox/Cetuximab (PR)
		11/05 + 04/06 liver metastasis	Gly12Ser	WT	07/06–09/06 Folfox/Cetuximab (PD)
		11/06 lung metastasis	Gly12Ser	WT	09/06-11/06 Folfiri/Avastin (PD)

10	M/77 y	11/05 primary CRC	Gly13Asp	WT	12/05–09/06 Folfox + Cetuximab (PD)
		07/06 lymph node	Gly13Asp	WT	10/06–02/07 Folfiri + Avastin (PD) dead 05/07

11	M/63 y	05/07primary CRC	WT	WT	05/07–07/08 Folfiri/Cetuximab (PR)
		01/08 liver metastasis	WT	WT	10/08–05/09 Folfox (PR)
		06/09 liver metastasis	WT	WT	

12	M/67 y	07/07 liver metastasis	WT	WT	08/07–04/08 Folfox/Cetuximab (PR)
		01/08 liver metastasis	WT	WT	

13	M/59 y	09/07 primary CRC	WT	WT	
		10/07 liver metastasis	WT	WT	11/07–03/08 Folfiri/Cetuximab (PR)
		05/08 liver metastasis	WT	WT	

14	M/61 y	07/02 primary CRC	WT	WT	10/07–07/08 Folfox/Cetuximab (PR)
		05/04 liver metastasis	WT	WT	
		07/08 peritoneal carcinosis	WT	WT	Since 12/08 Folfox (PR)

15	F/68 y	09/05 primary CRC	WT	V600E	10/05–02/06 Folfox (PD)
		05/06 soft tissue	WT	V600E	03/06–08/06 Folfiri/Cetuximab (PR)
		01/07 soft tissue	WT	V600E	01/07–05/07 Cetuximab/Irinotecon (PD) dead 06/07

16	M/75 y	07/00 primary CRC	Gly12Asp	WT	
		07/03 lung metastasis	Gly12Asp	WT	08/03–07/04 Folfox (PD)
		08/03 liver metastasis			08/04–08/05 Folfiri (PD)
		08/05 liver metastasis	Gly12Asp	WT	08/05–04/06 Folfiri/Cetuximab (PD) 04/06–02/07 Cetuximab+CPT-11(PD)02/07–04/07 Panitumumab (PD)
		04/07 ascites	Gly12Asp	WT	dead 05/07

17	M/69 y	06/02 primary CRC	Gly13Asp	WT	06/02–09/02 Folfox (PR) 06/03–12/03 Folfox (PR) 07/04–01/06 Folfiri (PD) 03/06–05/06 Cetuximab + CPT-11 (PD) 09/06–02/07 Folfox (PD)
		07/07 mesocolon metastasis	Gly13Asp	WT	dead 09/07

18	M/75 y	01/04 primary CRC	Gly12Asp	WT	06/07–10/07 Capecitabine/Cetximab (PD)
		01/08 peritoneal carcinosis	Gly12Asp	WT	dead 03/08

19	F/61 y	07/08 primary CRC	WT	WT	07/08–03/09 Folfiri/Cetuximab (PR)
		05/09 liver metastasis	WT	WT	

20	M/56 y	05/08 primary CRC	WT	WT	06/08-12/08 Folfox/Cetuximab (PR)
		06/09 lung metastasis	WT	WT	

21	M/72 y	09/08 primary CRC	WT	WT	10/08-02/09 Folfiri/Cetuximab (PR)
		01/09 liver metastasis	WT	WT	

Response evaluation (according to RECIST criteria): PD: progressive disease; SD: stable disease; PR: partial remission; CR: complete remission.
